# The importance of intersectoral factors in promoting equity-oriented universal health coverage: a multilevel analysis of social determinants affecting neonatal infant and under-five mortality in Bangladesh

**DOI:** 10.3402/gha.v9.29741

**Published:** 2016-02-12

**Authors:** Tanvir M. Huda, Tazeen Tahsina, Shams El Arifeen, Michael J. Dibley

**Affiliations:** 1 Sydney School of Public Health, The University of Sydney, Sydney, Australia; 2 Centre for Child and Adolescent Health, icddr,b, Dhaka, Bangladesh

**Keywords:** social determinants of health, inequality, inequity, universal health coverage, neonatal mortality, infant mortality, under-five mortality, health inequality, intersectoral action, Bangladesh

## Abstract

**Introduction:**

Health is multidimensional and affected by a wide range of factors, many of which are outside the health sector. To improve population health and reduce health inequality, it is important that we take into account the complex interactions among social, environmental, behavioural, and biological factors and design our health interventions accordingly.

**Objectives:**

This study examines mortality differentials in children of different age groups by key social determinants of health (SDH) including parental education and employment, mother's level of autonomy, age, asset index, living arrangements (utilities), and other geographical contextual factors (area of residence, road conditions).

**Design:**

We used data from the two rounds of Bangladesh Health and Demographic Survey, a nationally representative sample survey of the population residing in Bangladesh. Multilevel logistic models were used to study the impact of SDH on child mortality.

**Results:**

The study found that the mother's age, the education of both parents, the mother's autonomy to take decisions about matters linked to the health of her child, the household socio-economic conditions, the geographical region of residence, and the condition of the roads were significantly associated with higher risks of neonatal, infant, and under-five mortality in Bangladesh.

**Conclusion:**

The study findings suggest there are complex relationships among different SDH. Thus larger intersectoral actions will be needed to reduce disparities in child health and mortality and achieve meaningful progress towards equity-oriented universal health coverage.

## Introduction

One of the key targets for the sustainable development goals (SDGs) is to achieve universal health coverage (UHC), which aims to provide financial risk protection, access to quality essential healthcare services and access to safe, effective, quality and affordable essential medicines and vaccines for all (1). However, healthcare and health outcomes are often driven by social factors and affected not only by the access and use of healthcare services, but by an array of factors outside health sector including social, economic, political, and environmental factors (2). These factors, which contribute directly to health and disease as well as interacting with biological factors, are known as social determinants of health (SDH). Differences in the SDH largely explain the widespread health inequalities seen within and between countries (3). However, not all health inequalities are inequitable. Some health inequalities are unbiased because of biological phenomena (e.g. women live longer than men). *Health inequity* refers to those health inequalities that are unjust or unfair (4).

Bangladesh has set the ambitious goal of achieving UHC by 2032, as documented by the Bangladesh Health Care Financing Strategy (5). Over the past 44 years since independence, Bangladesh has made significant progress in the health sector. This progress is evident by the large reduction in under-five and infant mortality and increase in life expectancy at birth (6–9). Bangladesh's most remarkable achievement is its significant improvements in gender and socio-economic inequities over time. The *Lancet* series on Bangladesh (10–14) also affirmed the country's approach to equity. The success of Bangladesh has been attributed to the pluralistic health system, which pursues women-focused, equity-oriented, nationally targeted programs and positive social policies related to women's empowerment, widespread education, and mitigation of the effect of natural disasters.

Despite such progress in overall health and equity indicators, inequality in the coverage of different health services still remains a major concern especially for maternal and child health as reported by recent health and demographic surveys (6–9). For instance the 2011 Bangladesh Health and Demographic Survey (BDHS) reported that 74% of mothers from Khulna (in the southern part of the country) received antenatal care from a medically trained provider, compared to only 53% of mothers from Sylhet (in the northern part of the country). Similarly, 88% of mothers from the highest wealth quintile received antenatal care from a trained provider compared to only 35% of mothers from the lowest wealth quintile (9). The likelihood of receiving antenatal care from a medically trained provider also increases with the mother's education level and wealth status. Different survey findings also suggest there are strong differences in different health indicators as measured by self-perceived health or mortality (15–17).

Thus to improve the health of the population and reduce health inequalities it is crucial we take into account the role of SDH on health outcome and disease and design our interventions accordingly. Current health initiatives might address some of the determinants, but for more effective interventions much larger intersectoral actions and social participation are needed, with health policymakers playing key leadership roles.

However, given the common belief that access to health services is the only key to good health, strong evidence needs to be generated to identify the role of SDH on health and social well-being. This paper seeks to assess the impact of key social determinants on neonatal, infant, and under-five mortality in Bangladesh.

## Methods

The current study used data from the 2007 and 2011 BDHS (6, 9). The BDHS is a nationally representative sample survey of the population residing in private dwelling units in Bangladesh. The surveys used enumeration areas from the preceding national population and housing census as the primary sampling unit or cluster.

BDHS surveys used a two-stage cluster stratified sampling design. In 2007 there were 361 enumeration areas, or clusters, whereas in 2011 the number of clusters was increased to 600. In the second stage, a systematic sample of 30 households on average was selected per cluster to provide statistically reliable estimates of key demographic and health variables for the country as a whole, for urban and rural areas separately, and for each of the seven divisions. Reproductive histories were gathered from all married women aged 15–49 years. The survey asked each female respondent about all their births. We used births within the last 15 years, which gave us a total sample size of 40,651 live births.

### Ethical consideration

This study is based on an analysis of publicly available survey data with all identifier information removed. The survey was approved by the institutional review board of ICF Macro in Calverton, MD, USA. All study participants gave informed consent before participation and all information was collected confidentially.

### Conceptual framework and variables

**Fig. 1:**
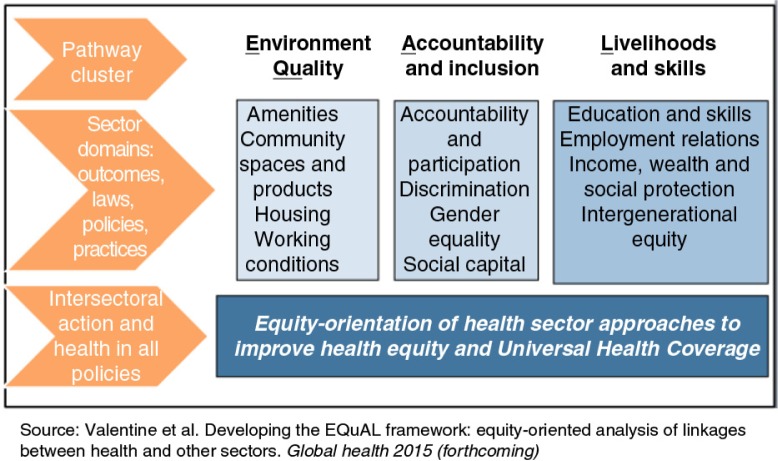
The EQuAL indicator and monitoring framework for health.

We used the 12 domains (i.e. income and poverty, knowledge and education, housing and infrastructure, travel, community and infrastructure, social protection and employment, early child development, gender norms, participation, registration, accountability, and discrimination) that were originally identified as part of a collaborative project with the World Health Organization (WHO) to monitor intersectoral factors that influence equity-oriented progress towards UHC and health equity (Fig. 1) (18, 19). According to the equity-oriented analysis of linkages between health and other sectors (EQuAL) framework^1^ the 12 domains were later refined and condensed into three broad domains – environmental quality, accountability and inclusion, and livelihoods and skills that remained compatible with the original domains (20). Among the 12 original domains we analysed data from seven domains, namely housing and infrastructure, income and poverty, knowledge and education, housing and infrastructure, travel, social protection and employment, and gender norms.

### Outcome variables

We examined neonatal mortality (the number of deaths of children under 1 month of age per 1,000 live births), infant mortality (the number of deaths of children under 1 year of age per 1,000 live births), and under-five mortality (the number of deaths of children under 5 years of age per 1,000 live births) as our dependent or outcome variables. The dependent variables in our analyses were categorical (1=death; 0=survival) and included all deaths in the specific age interval irrespective of the causes. The analysis of mortality examined neonatal, infant, and under-five mortality disaggregated by different SDH.

### Explanatory variables

We considered individual-, household-, and community-level covariates in our analysis. Individual-level covariates included the mother's age at birth, the mother's employment status in the last 12 months, both parents’ education, and the mother's participation in decision-making processes regarding child health. Household-level covariates included household access to safe drinking water, safe cooking and safe energy, and household assets score. The contextual, or community-level, variables included the condition of the main access road of the village, area of residence, and region. Age was self-reported and used as a categorical variable (less than 20, 20–34, and more than 34 years). Both mother's and father's education levels were divided into no education, primary, secondary, and higher levels of education. All variables except date of birth were categorical. Although the BDHS data set provided a household asset score, we recalculated the household wealth index excluding household access to safe water, clean energy for cooking, and electricity, since we intended to use these variables as separate covariates. The household wealth index was calculated using an inventory of household assets, which were weighted using the principal components analysis method (21).

### Statistical analysis: use of multilevel logistic regression

We used individual sampling weights to account for different sampling probabilities and different response rates. The household weight for a particular household used in DHS survey is the inverse of its household selection probability multiplied by the inverse of the household response rate of its household response rate group. The individual sampling weight was constructed by multiplying the household weight with the inverse of the individual response rate of his or her individual response rate group (6–9). Bivariate logistic regression was used to examine the association between the explanatory variables and the dependent outcomes. Only explanatory variables that were statistically significant (*p*<0.2) were incorporated into multivariable logistic regression. Since we included multiple births from the same mother, deaths of children from the same mother could be correlated because they shared the same risks associated with most determinants included in the analysis. Moreover DHS data are hierarchical (i.e. households are nested within clusters). We were concerned that such data could violate the assumption of independent observations used in logistic regression. Under such circumstances, standard errors are usually underestimated. Thus, we used multilevel modelling with three levels. The children (Level 1) nested within mothers (Level 2), who nested within clusters (Level 3); odds ratios were reported for each of the covariates. All disaggregated mortality rates were produced using the life table method (22). Stata 13 (StataCorp LP, College Station, TX, USA) was used for all statistical analyses.

## Results

We included 40,651 children born between 1997 and 2011, of which 15,997 children were born between 1997 and 2001, 15,846 between 2002 and 2006, and 8,800 between 2007 and 2011. Of the total children 51.4% were male, and the majority of births occurred in rural areas (68%). The highest percentage of births was from mothers who had no education (39%) and were aged 20–34 years at the time of birth (62%). The weighted frequency of births across the other determinants is presented in Table 1.

**Table 1:** Weighted frequency distribution of birth by different SDH in Bangladesh, 1997–2011

	1997–2001		2002–2006		2007–2011	
			
	*n*	%	*n*	%	*n*	%
Overall	15,997	100	15,854	100	8,800	100
Maternal employment status in last 12 months						
No outside work	11,984	75.0	12,578	79.3	7,809	88.7
Outside work	4,002	25.0	3,275	20.7	991	11.3
Mother's age at birth						
Less than 20 years	5,431	34.0	5,471	34.5	2,807	31.9
Between 20 and 34 years	9,841	61.6	9,489	59.9	5,617	63.8
More than 34 years	714	4.4	893	5.6	376	4.2
Autonomy (participation in child health-related decisions)^a^^b^^c^						
Do not participate	2,212	13.8	2,509	15.8	2,549	27.9
Participate	6,692	41.9	6,920	43.6	5,657	65.3
Mother's education						
No education	6,661	41.7	4,690	29.6	1,768	20.1
Primary level	5,295	33.1	5,201	32.8	2,699	30.7
Secondary level	3,388	21.2	5,094	32.1	3,696	42.0
Tertiary level	642	4.0	869	5.5	637	7.2
Housing/infrastructure (presence of three amenities)						
Yes	13,976	90.5	13,364	90.0	6,923	88.3
No	1,470	9.5	1,492	10.0	916	11.7
Household wealth index						
1 (poorest)	3,632	22.7	3,697	23.3	1,900	21.6
2	3,789	23.7	3,494	22.0	2,032	21.223.1
3	3,460	21.6	3,364		1,758	20.0
4	2,857	17.9	2,934	18.5	1,687	19.2
5 (richest)	2,248	14.1	2,365	14.9	1,423	16.2
Father's education						
No education	6,780	42.4	5,811	36.7	2,608	29.6
Primary level	4,356	27.2	4,542	28.7	2,556	29.0
Secondary level	3,399	21.3	3,819	24.1	2,543	28.9
Tertiary level	1,452	9.1	1,681	10.6	1,093	12.4
Area of residence						
Urban	3,396	21.2	3,441	21.7	1,997	22.7
Rural	12,590	78.8	12,413	78.3	6,802	77.3
Region						
Barisal	989	6.2	906	5.7	475	5.4
Chittagong	3,245	20.3	3,413	21.5	1,968	22.4
Dhaka	5,046	31.6	5,076	32.0	2,795	31.8
Khulna	1,654	10.3	1,511	9.5	817	9.3
Rajshahi	2,783	17.4	2,568	16.2	1,179	13.4
Rangpur	1,596	9.9	1,646	10.4	931	10.6
Sylhet	672	4.2	733	4.6	635	7.2
Main access road						
Good condition^d^	11,136	69.7	10,997	69.4	6,157	70.3
Poor condition	4,070	25.5	4,003	25.2	2,173	24.8
Others	780	4.8	853	5.4		

a 7,083 missing values for this variable in 1997–2001.

b 6,425 missing values for this variable in 2002–2007.

c 594 missing values for this variable in 1997–2001.

d Indicates roads built of brick or modern materials and accessible in all seasons.

Tables 2–4 show neonatal, infant, and under-five mortality rates according to different SDH in 5-year time periods. All three mortality rates showed similar associations and trends across the different time periods. Children born to younger mothers (less than 20 years of age), mothers with no or minimal education, and mothers who had no participation in child health-related decisions had higher rates of neonatal, infant, and under-five mortality. Children born to working mothers between 2007 and 2011 had higher rates of neonatal, infant, and under-five mortality but this was not the case in children born in the two earlier time periods.

**Table 2:** Neonatal mortality rate (NMR) (deaths in children 0–30 days per 1,000 live births), with 95% confidence interval disaggregated by SDH and intervals of year of birth in Bangladesh, 1997–2011

	1997–2001	2002–2006	2007–2011
			
	NMR (95% CI)	NMR (95% CI)	NMR (95% CI)
Maternal employment status in last 12 months			
No outside work	44.3 (40.8–48.1)	38.4 (35.2–41.9)	31.2 (27.5–35.3)
Outside work	40.4 (34.6–47.2)	38.2 (32.1–45.5)	45.1 (33.9–60.0)^a^
Mother's age at birth			
Less than 20 years	57.4 (51.5–64.0)	50.7 (45.2–57.0)	42.9 (35.9–51.3)
Between 20 and 34 years	35.2 (31.7–39.0)	32.3 (29.0–36.0)	27.4 (23.4–32.0)
More than 34 years	52.3 (38.6–70.6)	30.9 (21.4–44.4)	40.3 (24.9–64.9)
Autonomy (participation in child health-related decisions)^a^			
Do not participate	47.7 (39.3–57.8)	48.5 (40.6–57.9)	45.4 (37.8–54.5)
Participate	42.0 (37.5–47.1)	36.6 (32.5–41.3)	26.6 (22.7–33.1)
Mother's education			
No education	47.2 (42.2–52.7)	46.2 (40.5–52.8)	32.4 (24.9–42.1)
Primary level	45.6 (40.3–51.5)	40.5 (35.5–46.3)	35.2 (28.8–43.0)
Secondary level	37.0 (31.3–43.7)	33.7 (29.1–39.0)	33.5 (28.1–39.8)
Tertiary level	25.9 (16.8–39.9)	17.3 (11.0–27.4)	21.1 (12.8–34.7)
Housing/infrastructure (presence of three amenities)			
Yes	31.0 (23.5–40.9)	25.8 (19.1–34.7)	22.8 (14.9–34.7)
No	44.6 (41.3–48.1)	40.0 (36.8–43.5)	32.7 (28.7–37.2)
Household wealth index			
1 (poorest)	51.7 (44.8–59.7)	45.0 (38.5–52.5)	33.3 (26.0–42.7)
2	45.6 (39.3–53.0)	42.0 (35.7–49.4)	40.2 (32.2–50.2)
3	43.0 (36.6–50.5)	39.7 (33.5–47.0)	31.3 (24.0–40.8)
4	45.0 (38.1–53.0)	35.9 (29.8–43.1)	34.8 (27.2–44.5)
5 (richest)	28.5 (22.9–35.5)	27.5 (22.1–34.3)	23.0 (16.7–31.7)
Father's education			
No education	46.7 (41.8–52.2)	48.8 (43.4–54.8)	35.7 (29.0–43.9)
Primary level	41.9 (36.4–48.2)	39.2 (33.9–45.2)	36.9 (30.2–45.0)
Secondary level	44.4 (38.1–51.7)	29.6 (24.7–35.4)	30.6 (24.6–38.0)
Tertiary level	31.2 (23.7–40.9)	24.6 (18.5–32.7)	22.9 (15.8–33.3)
Area of residence			
Urban	40.7 (35.6–46.5)	32.4 (27.9–37.7)	32.8 (26.7–40.2)
Rural	44.6 (40.9–48.6)	41.2 (37.6–45.2)	32.8 (28.6–37.6)
Region			
Barisal	29.8 (21.7–40.7)	27.0 (20.6–35.5)	37.2 (26.9–51.5)^b^
Chittagong	33.9 (27.9–41.1)	35.2 (29.2–42.3)	23.4 (17.3–31.8)
Dhaka	40.5 (34.0–48.2)	32.5 (26.7–39.6)	35.8 (27.5–46.5)
Khulna	45.0 (36.8–55.1)	31.4 (24.4–40.5)	32.8 (23.4–45.8)
Rajshahi	47.7 (39.7–57.2)	46.6 (38.5–56.3)	29.7 (21.1–41.7)
Rangpur	57 (48.4–67)	46.5 (38.8–55.6)	30.7 (22.1–42.5)
Sylhet	47.3 (37.4–59.7)	53.8 (43.6–66.3)	42.7 (33.0–55.1)
Main access road			
Good condition^c^	41.8 (38.3–45.6)	35.5 (32.2–39.1)	32.7 (28.6–37.5)
Poor condition	48.7 (42.2–56.3)	48.4 (41.9–56.0)	34.8 (27.8–43.4)

a The number of births are small in these categories and may not be adequate to measure mortality precisely.

b The sample size was small (*n*=946) and insufficient to calculate the mortality rate.

c Indicates roads built of brick or modern materials and accessible in all seasons.

**Table 3:** Infant mortality rate (IMR) (deaths in children <12 m of age per 1,000 live births), with 95% confidence interval, disaggregated by SDH and intervals of year of birth in Bangladesh, 1997–2011

	1997–2001	2002–2006	2007–2011
			
	IMR (95% CI)	IMR (95% CI)	IMR (95% CI)
Maternal employment status in last 12 months			
No outside work	66.9 (62.6–71.5)	52.9 (49.1–56.9)	39.5 (35.3–44.2)
Outside work	61.4 (54.2–69.5)	52.5 (45.3–60.8)	65.5 (51.6–83.0)^a^
Mother's age at birth			
Less than 20 years	82.5 (75.4–90.3)	65.7 (59.3–72.7)	54.2 (46.2–63.5)
Between 20 and 34 years	55.2 (50.9–59.8)	46.0 (42.0–50.4)	36.3 (31.6–41.6)
More than 34 years	83.7 (66.1–105.6)	49.8 (37.4–66.1)	50.9 (33.1–77.8)
Autonomy (participation in child health-related decision)^a^			
Do not participate	70.8 (60.6–82.7)	64.9 (55.7–75.5)	59.8 (51.0–70.1)
Participate	63.6 (58.1–69.7)	49.8 (45.0–55.2)	34.1 (29.6–39.1)
Mother's education			
No education	75.1 (68.9–81.9)	71.0 (63.8–78.8)	54.9 (44.8–67.2)
Primary level	70.2 (63.6–77.3)	54.8 (48.9–61.3)	44.0 (36.8–52.7)
Secondary level	48.8 (42.2–56.4)	41.1 (36.0–46.9)	39.9 (34.0–46.8)
Tertiary level	33.7 (23.1–49.1)	22.2 (14.8–33.2)	21.1 (12.8–34.7)
Housing/infrastructure (presence of three amenities)			
Yes	45.6 (36.4–57.1)	36.8 (28.7–47.1)	29.7 (20.5–43.1)
No	67.9 (63.9–72.2)	55.1 (51.3–59.1)	42.9 (38.3–48.0)
Household wealth index			
1 (poorest)	81.4 (72.7–91.2)	65.3 (57.5–74.1)	45.4 (36.6–56.2)
2	68.8 (60.9–77.6)	58.4 (50.9–66.9)	55.7 (46.0–67.4)
3	71.2 (62.9–80.5)	54.4 (47.1–62.8)	37.7 (29.6–48.0)
4	60.5 (52.6–69.6)	49.0 (41.9–57.2)	42.0 (33.4–52.6)
5 (richest)	40.6 (33.8–48.7)	33.6 (27.5–40.9)	29.8 (22.5–39.5)
Father's education			
No education	76.5 (70.2–83.2)	70.3 (63.8–77.4)	48.5 (40.6–57.9)
Primary level	64.7 (57.8–72.3)	52.9 (46.8–59.8)	50.5 (42.5–59.9)
Secondary level	59.3 (52.0–67.5)	39.6 (33.9–46.2)	38.1 (31.2–46.4)
Tertiary level	38.6 (30.2–49.3)	28.9 (37.6–22.2)	22.9 (15.8–33.3)
Area of residence			
Urban	67.7 (63.1–72.6)	56.4 (52.2–60.9)	42.4 (37.5–47.8)
Rural	61.1 (54.8–68.0)	45.2 (39.9–51.3)	42.9 (35.8–51.3)
Region			
Barisal	43.1 (33.3–55.8)	40.2 (32.1–50.2)	49.1 (36.8–65.2)^b^
Chittagong	53.1 (45.5–61.9)	48.3 (41.3–56.5)	32.6 (25.0–42.4)
Dhaka	62.1 (53.9–71.4)	46.8 (39.7–55.0)	43.1 (33.9–54.8)
Khulna	66.1 (56.0–77.9)	41.2 (33.0–51.3)	38.1 (27.9–52.0)
Rajshahi	65.0 (55.7–75.8)	56.8 (47.8–67.4)	42.2 (31.6–56.4)
Rangpur	81.3 (71.0–93.0)	68.1 (58.7–78.9)	40.0 (30.0–53.2)
Sylhet	79.8 (66.8–95.2)	72.6 (60.7–86.7)	55.6 (44.4–69.6)
Main access road			
Good condition^c^	62.5 (67.1–58.2)	48.9 (45.1–53.1)	43.0 (38.1–48.4)
Poor condition	73.4 (65.3–82.4)	65.8 (58.1–74.4)	41.8 (38.3–45.6)

a The number of births in these categories is low and may not be adequate to measure mortality precisely.

b The sample size was small (*n*=946) and insufficient to calculate the mortality rate.

c Indicates roads built of brick or modern materials and accessible in all seasons.

**Table 4:** Mortality rate among children under five (deaths in children <5 years per 1,000 live births), with 95% confidence interval, disaggregated by SDH and intervals of year of birth in Bangladesh, 1997–2011

	1997–2001	2002–2006	2007–2011
			
	Under-five mortality (95% CI)	Under-five mortality (95% CI)	Under-five mortality (95% CI)
Maternal employment status in last 12 months			
No outside work	84.2 (79.4–89.3)	66.0 (61.8–70.5)	47.9 (43.0–53.3)
Outside work	79.2 (71.0–88.3)	63.4 (55.3–72.6)	71.0 (56.4–89.3)^a^
Mother's age at birth			
Less than 20 years	99.9 (92.1–108.3)	77.5 (70.5–85.1)	61.5 (52.5–71.8)
Between 20 and 34 years	72.0 (67.1–77.2)	58.6 (54.0–63.6)	45.2 (39.7–51.5)
More than 34 years	109.8 (89.6–134.2)	68.6 (53.6–87.6)	50.9 (33.1–77.8)
Autonomy (participation in child health-related decisions)^a^^b^^c^			
Do not participate	91.6 (79.9–104.8)	78.7 (68.5–90.2)	67.4 (57.6–78.8)
Participate	78.8 (72.7–85.6)	62.3 (56.9–68.3)	42.5 (37.2–48.4)
Mother's education			
No education	99.8 (92.6–107.4)	85.9 (78.0–94.6)	64.8 (53.4–78.5)
Primary level	86.5 (79.3–94.4)	70.2 (63.5–77.6)	52.5 (44.2–62.3)
Secondary level	58.0 (50.8–66.2)	49.8 (44.1–56.2)	48.1 (41.1–56.3)
Tertiary level	37.6 (26.3–53.6)	30.1 (21.1–42.8)	21.1 (12.8–34.7)
Housing/infrastructure (presence of three amenities)			
Yes	55.7 (45.5–68.2)	46.9 (37.6–58.5)	36.6 (25.7–52.0)
No	86.1 (81.6–90.0)	67.9 (63.7–72.4)	50.3 (45.1–56.1)
Household wealth index			
1 (poorest)	102.6 (92.9–113.3)	76.2 (67.7–85.7)	54.1 (43.9–66.5)
2	90.7 (81.7–100.7)	72.7 (64.2–82.2)	64.0 (53.1–77.1)
3	91.2 (81.9–101.6)	64.2 (56.1–73.3)	46.2 (36.5–58.4)
4	74.7 (65.9–84.7)	63.0 (54.8–72.4)	51.8 (41.8–64.1)
5 (richest)	47.9 (40.5–56.6)	40.7 (34.0–48.8)	34.1 (25.9–44.8)
Father's education			
No education	98.0 (91.0–105.6)	85.9 (78.7–93.7)	58.3 (49.2–69.1)
Primary level	84.5 (76.7–93.1)	66.3 (59.3–74.1)	59.1 (50.2–69.7)
Secondary level	71.3 (63.4–80.3)	49.6 (43.1–57.1)	45.7 (37.6–55.5)
Tertiary level	44.9 (35.8–56.2)	36.8 (29.0–46.5)	26.0 (18.0–37.5)
Area of residence			
Urban	87.0 (81.8–92.4)	70.7 (65.9–75.8)	51.1 (45.4–57.5)
Rural	74.6 (67.7–82.1)	54.5 (48.6–61.2)	49.1 (41.3–58.4)
Region			
Barisal	82.8 (71.6–95.7)	56.0 (46.2–67.8)	60.2 (46.0–78.6)^b^
Chittagong	73.3 (64.4–83.4)	69.0 (60.4–78.8)	43.5 (34.1–55.3)
Dhaka	79.9 (70.7–90.3)	55.0 (47.3–64.0)	50.9 (40.1–64.5)
Khulna	74.6 (63.9–87.0)	49.4 (40.3–60.5)	39.9 (29.2–54.4)
Rajshahi	79.3 (69.0–91.1)	64.4 (54.7–75.7)	50.1 (37.9–66.2)
Rangpur	100.1 (88.7–112.9)	81.2 (70.8–93.0)	44.8 (33.7–59.4)
Sylhet	98.9 (84.4–115.6)	86.2 (73.2–101.3)	64.8 (52.0–80.5)
Main access road			
Good condition^c^	78.1 (73.4–83.2)	60.8 (56.5–65.5)	50.1 (44.6–56.3)
Poor condition	96.3 (87.2–106.4)	79.4 (71.0–88.9)	41.8 (38.3–45.6)

a The number of births in these categories is low and may not be adequate to measure mortality precisely.

b The sample size was small (*n*=946) and insufficient to calculate mortality rate.

c Indicates roads built of brick or modern materials and accessible in all seasons.

The mortality rates were also disaggregated according to household-level factors. All mortality rates were found to be lower in children born to women whose partner had a higher level of education and came from the wealthiest households and from households with access to safe water, clean energy for cooking, and electricity.

As seen from the community-level factors in Tables 2–4, children born in rural communities or living in areas with poor main access roads that were not accessible in all seasons had a higher risk of neonatal, infant, and under-five mortality. However, the mortality rates were similar for children from rural versus urban communities for children who were born in 2007 or later. The mortality rates also differed across regions. Sylhet had the highest neonatal, infant, and under-five mortality rates in children born between 2002 and 2011. Rangpur had the highest rates of mortality in children born earlier than 2002.

As expected, the mortality rates across all categories decreased over the time and the pattern of association was similar in all three time periods except for the autonomy variable and Barisal region.

The unadjusted and adjusted ORs of the potential factors associated with neonatal, infant, and under-five mortality are presented in Tables 5–7. The odds of neonatal, infant, or under-five mortality varied greatly according to geographical region, with significantly higher adjusted odds of death in all mortality age groups in Rangpur and Sylhet compared to Barisal.

**Table 5:** Odds ratio for mortality of children <1 m of age for SDH determined by multilevel multivariate analysis in Bangladesh, 1997–2011^a^

	Crude OR (95% CI)	*P*	Adjusted OR (95% CI)^b^	*P*
Maternal employment status in last 12 months				
No outside work	Ref.			
Outside work	1.10 (0.99–1.23)	0.00**	1.05 (0.92–1.20)	0.48
Mother's age at birth				
Less than 20 years	Ref.			
Between 20 and 34 years	0.62 (0.56–0.68)	0.00**	0.60 (0.53–0.66)	0.00**
More than 34 years	0.7 (0.60–0.93)	0.01**	0.74 (0.58–0.95)	0.02**
Autonomy (participation in child health-related decisions)				
Do not participate	Ref.			
Participate	0.74 (0.65–0.85)	0.00**	0.76 (0.65–0.90)	0.00**
Mother's education				
No education	Ref.			
Primary level	0.91 (0.81–1.01)	0.09*	0.95 (0.82–1.09)	0.44
Secondary level	0.72 (0.63–0.81)	0.00**	0.83 (0.69–0.99)	0.04**
Tertiary level	0.42 (0.31–0.55)	0.00**	0.63 (0.42–0.93)	0.02**
Housing/infrastructure (presence of three amenities)				
Yes	Ref.			
No	0.65 (0.53–0.79)	0.00**	0.94 (0.742–1.23)	0.66
Household wealth index				
1 (poorest)	Ref.			
2	0.95 (0.83–1.08)	0.47	0.97 (0.83–1.14)	0.69
3	0.84 (0.73–0.97)	0.00**	0.90 (0.76–1.06)	0.21
4	0.83 (0.72–0.96)	0.00**	0.91 (0.76–1.09)	0.30
5 (richest)	0.56 (0.47–0.67)	0.00**	0.68 (0.53–0.88)	0.00**
Father's education				
No education	Ref.			
Primary level	0.82 (0.73–0.91)	0.00**	0.84 (0.73–0.97)	0.02**
Secondary level	0.77 (0.68–0.86)	0.00**	0.92 (0.78–1.08)	0.31
Tertiary level	0.54 (0.44–0.64)	0.00**	0.89 (0.67–1.17)	0.40
Area of residence				
Urban	Ref.			
Rural	1.16 (1.03–1.29)	0.01**	0.92 (0.79–1.06)	0.26
Region				
Barisal	Ref.			
Chittagong	0.96 (0.77–1.19)	0.74	0.94 (0.75–1.20)	0.63
Dhaka	1.11 (0.90–1.37)	0.32	1.13 (0.89–1.42)	0.32
Khulna	1.16 (0.92–1.45)	0.20	1.13 (0.89–1.45)	0.32
Rajshahi	1.40 (1.13–1.74)	0.00**	1.30 (1.03–1.64)	0.03**
Rangpur	1.48 (1.19–1.83)	0.00**	1.44 (1.14–1.82)	0.00**
Sylhet	1.42 (1.11–1.81)	0.00**	1.58 (1.21–2.07)	0.00**
Main access road				
Good condition	Ref.			
Poor condition	1.22 (1.08–1.38)	0.00**	1.16 (1.01–1.34)	0.03**

a A multilevel logistic regression with three levels was done to adjust cluster effect and mother effect.

b aOR, adjusted OR, adjusted for date of birth and all other variables listed in the tables.

* *p*<0.1;

** *p*<0.05.

**Table 6:** Odds ratio for mortality of children <12 m of age for SDH determined by multilevel multivariate analysis in Bangladesh, 1997–2011^a^

	Crude OR (95% CI)	*P*	Adjusted OR (95% CI)^b^	*P*
Maternal employment status in last 12 months				
No outside work	Ref.			
Outside work	1.15 (1.05–1.26)	0.00**	1.05 (0.94–1.18)	0.36
Mother's age at birth				
Less than 20 years	Ref.			
Between 20 and 34 years	0.66 (0.61–0.72)	0.00**	0.64 (0.58–0.70)	0.00
More than 34 years	0.82 (0.68–0.98)	0.03**	0.75 (0.61–0.92)	0.01
Autonomy (participation in child health-related decisions)				
Do not participate	Ref.			
Participate	0.76 (0.68–0.85)	0.00**	0.79 (0.69–0.90)	0.00**
Mother's education				
No education	Ref.			
Primary level	0.83 (0.76–0.91)	0.00**	0.89 (0.80–1.00)	0.05**
Secondary level	0.57 (0.51–0.62)	0.00**	0.72 (0.62–0.83)	0.00**
Tertiary level	0.31 (0.24–0.40)	0.00**	0.53 (0.38–0.75)	0.00**
Housing/infrastructure (presence of three amenities)				
Yes	Ref.			
No	0.63 (0.53–0.74)	0.00**	0.99 (0.79–1.24)	0.94
Household wealth index				
1 (poorest)	Ref.			
2	0.88 (0.79–0.98)	0.02**	0.89 (0.78–1.02)	0.09*
3	0.81 (0.72–0.90)	0.00**	0.89 (0.77–1.02)	0.08*
4	0.73 (0.64–0.82)	0.00**	0.85 (0.73–0.98)	0.03**
5 (richest)	0.48 (0.41–0.55)	0.00**	0.64 (0.52–0.80)	0.00**
Father's education				
No education	Ref.			
Primary level	0.81 (0.73–0.89)	0.00**	0.89 (0.79–1.00)	0.04**
Secondary level	0.66 (0.60–0.74)	0.00**	0.87 (0.76–1.00)	0.05**
Tertiary level	0.42 (0.35–0.50)	0.00**	0.79 (0.62–1.00)	0.05**
Area of residence				
Urban	Ref.			0.12
Rural	1.18 (1.07–1.30)	0.00**	0.91 (0.81–1.03)	0.10*
Region				
Barisal	Ref.			
Chittagong	0.90 (0.75–1.08)	0.26	0.91 (0.75–1.10)	0.32
Dhaka	1.05 (0.87–1.25)	0.61	1.05 (0.87–1.27)	0.61
Khulna	0.99 (0.82–1.20)	0.94	1.01 (0.83–1.24)	0.90
Rajshahi	1.16 (0.97–1.40)	0.10*	1.06 (0.87–1.28)	0.59
Rangpur	1.39 (1.16–1.66)	0.00**	1.34 (1.11–1.62)	0.00**
Sylhet	1.33 (1.08–1.63)	0.00**	1.51 (1.21–1.88)	0.00**
Main access road				
Good condition	Ref.			
Poor condition	1.21 (1.09–1.33)	0.00	1.14 (1.02–1.28)	0.03

a A multilevel logistic regression with three levels was done to adjust cluster effect and mother effect.

b aOR, adjusted OR, adjusted for date of birth and all other variables listed in the tables.

* *p*<0.1;

** *p*<0.05.

**Table 7:** Odds ratio for mortality of children under five for SDH determined by multilevel multivariate analysis in Bangladesh, 1997–2011^a^

	Crude OR (95% CI)	*P*	Adjusted OR (95% CI)^b^	*P*
Maternal employment status in last 12 months				
No outside work	Ref.			
Outside work	1.18 (1.08–1.28)	0.00**	1.04 (0.95–1.14)	0.40
Mother's age at birth				
Less than 20 years	Ref.			
Between 20 and 34 years	0.73 (0.67–0.78)	0.00**	0.72 (0.67–0.78)	0.00**
More than 34 years	0.90 (0.76–1.05)	0.19	0.83 (0.71–0.99)	0.04**
Autonomy (participation in child health-related decisions)				
Do not participate	Ref.			
Participate	0.79 (0.71–0.88)	0.00**	0.81 (0.73–0.91)	0.00**
Mother's education				
No education	Ref.			
Primary level	0.78 (0.71–0.84)	0.00**	0.86 (0.78–0.94)	0.01**
Secondary level	0.50 (0.45–0.55)	0.00**	0.69 (0.61–0.78)	0.00**
Tertiary level	0.27 (0.21–0.34)	0.00**	0.51 (0.37–0.68)	0.00**
Housing/infrastructure (presence of three amenities)				
Yes	Ref.			
No	0.63 (0.54–0.73)	0.00**	1.06 (0.88–1.28)	0.53
Household wealth index				
1 (poorest)	Ref.			
2	0.90 (0.81–0.99)	0.00**	0.93 (0.83–1.03)	0.14
3	0.81 (0.73–0.89)	0.00**	0.89 (0.80–1.00)	0.04**
4	0.70 (0.63–0.78)	0.00**	0.84 (0.74–0.95)	0.01**
5 (richest)	0.45 (0.39–0.51)	0.00**	0.62 (0.51–0.74)	0.00**
Father's education				
No education	Ref.			
Primary level	0.79 (0.73–0.86)	0.00**	0.91 (0.84–1.00)	0.06*
Secondary level	0.62 (0.56–0.68)	0.00**	0.85 (0.76–0.96)	0.00**
Tertiary level	0.39 (0.33–0.45)	0.00*	0.79 (0.65–0.97)	0.02**
Area of residence				
Urban	Ref.			
Rural	1.22 (1.12–1.33)	0.00**	0.93 (0.84–1.03)	0.14
Region				
Barisal	Ref.			
Chittagong	0.96 (0.82–1.12)	0.60	0.96 (0.82–1.00)	0.64
Dhaka	0.98 (0.83–1.14)	0.77	0.97 (0.82–1.10)	0.68
Khulna	0.85 (0.71–1.00)	0.06*	0.85 (0.72–1.00)	0.10*
Rajshahi	1.05 (0.89–1.23)	0.59	0.96 (0.81–1.10)	0.61
Rangpur	1.28 (1.08–1.50)	0.00**	1.19 (1.01–1.30)	0.03**
Sylhet	1.20 (1.00–1.44)	0.05**	1.32 (1.09–1.52)	0.00**
Main access road				
Good condition	Ref.			
Poor condition	1.21 (1.10–1.33)	0.00**	1.14 (1.03–1.26)	0.00**

a A multilevel logistic regression with three levels was done to adjust cluster effect and mother effect.

b aOR, adjusted OR, adjusted for date of birth and all other variables listed in the tables.

* *p*<0.1;

** *p*<0.05.

Children of all ages who were living in areas with main roads in poor condition had around 21% higher odds of dying than children living in areas with roads in good condition. No significant differences in odds of death were found between rural and urban children for all mortality age groups.

Neonates, infants, and children under the age of five who were born to mothers from the wealthiest households had 30–38% lower odds of dying than those who were born to mothers from the poorest households. Wealth had a greater impact on mortality in the later years of life.

Maternal education played a more significant role than the education of the father. The risk of neonatal mortality was reduced by 37% in neonates whose mothers had tertiary-level education compared to neonates from mothers with no education. The risk was much lower for infant mortality (aOR 0.54) and under-five mortality (aOR 0.50). Similar to maternal education, paternal education was also a strong predictor for premature death in children in Bangladesh. Children from fathers who had tertiary-level education were 15–19% less likely to die prematurely during the neonatal period, infancy, or before the age of five. Children who had a younger mother (less than 20 years) during their birth were more likely to die prematurely before the age of five, whereas children from mothers aged 35–49 years at the time of birth had the least chance of dying in either the neonatal period, infancy, or before the age of 5 years. Women's decision-making power was also found to be an independent predictor. Mothers who had a voice in child health-related decisions were less likely to have a child die before the age of 1 month. The association was similar for infant and under-five deaths.

## Discussion

Our study reported that the odds ratio for childhood mortality was highest among young mothers, parents with lower education, mothers who lacked power to take decisions about matters linked to the health of their children, and those from lower socio-economic background. Furthermore children in Bangladesh who reside in communities with poor road connections and who are from selected regions are also more likely to die prematurely. The effects of other social determinants included in the study, such as mothers’ employment status and housing infrastructure, were not significant.

The current study showed that children born to mothers aged 20–34 years have the least risk of dying prematurely. Controlling for other variables in multivariable logistic analysis, maternal age remained a significant predictor for all six models in the study. Pregnancy in early age can be life-threatening to both the mother and the child. Younger mothers have an increased risk of maternal mortality. Early pregnancy also often disrupts education, which has a profound indirect effect on the health of both mother and child (23). A young mother also has less autonomy and decision-making authority. They sometimes hide their pregnancy and delay prenatal care. Several studies have found younger mothers to be a risk factor for child deaths. A study in India reported that increases in the mother's age corresponded with a reduction in child mortality (24). Another study in India reported the risk for both infant mortality (OR 1.50, 1.30 to 1.73) and child mortality (2.10, 1.31 to 3.38) were higher for children born to a woman married before age 18 (25). Similarly a study in Nigeria reported a significantly higher risk of neonatal deaths for neonates born to younger mothers (<20 years) [hazard ratio (HR)=4.07; 95% confidence interval (CI)=2.83–5.86) than neonates born to mothers aged 30–39 years (26). Other studies in Ethiopia and elsewhere have reported similar findings (27, 28).

The current study observed that children born to mothers with secondary- or tertiary-level education had a significantly lower risk of mortality than those born to mothers with no education or primary-level education. The effect of education on health has long been established as a major contributor to health advancements. Education has both direct and indirect effects on health. An educated mother is likely to have knowledge on the benefits of healthy behaviour and also has better access to healthcare services than an uneducated mother. Education also improves health indirectly through improved employment opportunities and income. A woman's education is particularly important as it not only affects her own health but also the health of her children and family. Different studies have shown strong relationships between education and various health outcomes, especially with child mortality, as women's education allows access to information about safe pregnancy and better child healthcare (16). A study in rural India found that the odds of neonatal deaths were lower for neonates born to mothers with secondary-level education and in addition that there was a progressive reduction in the odds of neonatal deaths as the level of fathers’ education increased (29). A study in Nigeria reported that children born to mothers with no formal education was significantly associated with post-neonatal, infant, child, and under-five mortality (30). Other studies in India and Bangladesh also showed a significant relationship between neonatal, infant, and under-five child mortality and maternal education (31–34).

In our study children of mothers who participated in the decision-making process regarding matters linked to the health of their child had a 19–24% lower risk of dying than children of mothers who had no decision-making power. Women's empowerment is an important determinant of access to healthcare services. Women who have greater decision-making authority tend to utilise healthcare services more for themselves and for their children than those who have little or no decision-making authority. A study in Nepal reported that infants of women who were involved in decision-making regarding their own healthcare had a 25% lower chance of dying than did infants whose mothers who were not involved in healthcare decisions (35). A study in Ghana reported financial autonomy of mothers to be significantly associated with under-five mortality (36).

The current study shows that children born to mothers from households with lower socio-economic status had a higher risk of neonatal mortality compared with those from households with higher socio-economic status. This finding is consistent with previous studies (27, 31, 32). Poverty and poor health are very closely linked. Studies have shown that the health of children from poor and middle-income households is notably worse than that of children in wealthier households. The relationship between poverty and health outcomes is multifaceted and often bidirectional. Poverty results in deficits of food, housing, sanitation, and safe drinking water. All of these have direct effects on health outcomes. The poor also have less use of health services. On the other hand, ill health can be a catalyst for poverty. A study in Nepal also found that inequalities in risk of neonatal mortality exist in relation to wealth status (37). In Nigeria, a study using the Cox proportional models showed that infants born to mothers from poor households (HR=1.40; CI=1.10–1.78) had a higher risk of infant mortality than children born in rich households (30). Similarly, for under-five mortality children from poor households were more likely to die within 59 months of life as compared with those from rich households (HR=1.43; CI=1.17–1.76) (30). A study conducted in urban India found that non-poor children had lower under-five mortality compared to the poor (38).

Our study used a proxy indicator such as condition of the main access road to assess the association between mortality and travel barriers to access to healthcare. Travel is considered to be one of the important barriers to utilisation of healthcare services, especially those directed towards women and child health (39). Long distance from the health facility influences the decision for care-seeking to a great extent. With long distances to travel to reach healthcare centres, the indirect costs can be substantial (40). In addition a long travel time also results in loss of working hours for the accompanying person. Studies have found that the poor are more sensitive to travel time to healthcare centre than their counterparts who are better-off (41). The condition of the main access road was found to be an independent predictor of neonatal, infant, and under-five mortality (41). A study in rural Burkina Faso reported distance to health facilities as a risk factor for both infant and under-five child deaths (42). In Madagascar the odds of infant mortality were found to be 42% higher among those living further than 5 km from a health centre compared to those living within 1.5–3.0 km (43).

Similar to our findings, other studies have also reported geographical variation in child mortality. A study in Ethiopia reported some regions had a 54 to 88% higher risk of death than other regions (28). These geographical variations were probably due to differences in culture and health-related behaviours of the population, but are also likely to reflect differences in health system performance. However, different studies reported contrasting findings on the impact of area of residence on child mortality. A study in Iran did not find any differences in infant mortality between urban and rural areas (44). However a study in Nigeria reported living in rural areas as a risk factor for post-neonatal, infant, and under-five mortality (30).

Our study did not report women's employment status as a risk factor, though other studies have shown a negative relationship between the mother's employment status and child mortality. A study in rural India reported that the odds of neonatal death were lower for infants born to unemployed mothers (OR=0.89) (29). A negative association was also found with infant and under-five mortality and female labour force participation in India (45). In addition our study did not find any significant association between child mortality and housing infrastructure as measured by access to safe water, clean fuel, and electricity; however, several other studies have established strong associations between poor health and poor housing and housing infrastructure (16, 17, 33, 45).

Health outcomes are socially determined. Health sector interventions alone are not sufficient to improve the health of the population and reduce health inequalities. Public policies that integrate health, social, and economic actions are needed to improve SDH. Researchers and program specialists need to generate enough evidence depicting the role of SDH on health disparity. Such evidence is required to convince policymakers, especially those from non-health sectors. However, there has been no study in Bangladesh that evaluated the impact of SDH on health outcomes, particularly on child mortality. Analysing a nationally representative dataset, this paper investigates the role of social determinants, including socio-economic position as well as geographical contextual factors, on neonatal, infant, and under-five mortality in all districts in Bangladesh. This study aims to provide enough evidence to policymakers in the country regarding the need for a comprehensive multisectoral approach to tackle health inequalities.

### Strengths and limitations

A major strength of our study is our use of nationally representative sample survey data because it gave us the opportunity to examine the geographical or regional variations in neonatal, infant, and under-five mortality. Because we have included all children born within the 15 years preceding the survey, there is a chance of recall bias, which could affect the internal validity of the study. However, as we have only used SDH as predictor variables and did not include any explanatory variables that require recollection of events from the past, we believe there would be limited recall bias and hence no effect to the internal validity. The BDHS only interviewed surviving mothers, which was one of the limitations of the current study. It may have led to an underestimate of the neonatal mortality rate, because of the association of neonatal deaths with maternal deaths, and could also have led to underestimation of the effect of some of the associated factors. Moreover some variables were not child-specific (e.g. socio-economic condition, education, condition of roads); as our data were cross-sectional, they only represented the most recent conditions. There is a possibility that socio-economic and other conditions may have changed over the period for some individuals and households.

## Conclusion

Our study suggests that social determinants play a strong role in child health and mortality. It is extremely important that we carefully plan, invest, and monitor not only the proximal determinants of child health (i.e. the biological and behavioural risk factors) but also the distal factors, which are strong determinants of neonatal, infant, and under-five mortality. Effective intersectoral actions will be needed in order to achieve meaningful progress towards equity-oriented UHC.

## Footnotes

1 More information about the EQuAL framework will be available in a forthcoming WHO report titled *Monitoring Health Determinants for Equity* (in preparation).
